# Altered cortico-striatal crosstalk underlies object recognition memory deficits in the sub-chronic phencyclidine model of schizophrenia

**DOI:** 10.1007/s00429-017-1393-3

**Published:** 2017-03-14

**Authors:** Aman Asif-Malik, Daniel Dautan, Andrew M. J. Young, Todor V. Gerdjikov

**Affiliations:** 1 Department of Neuroscience, Psychology and Behaviour, University of Leicester, Leicester, LE1 9HN UK; 2 Center for Molecular and Behavioral Neuroscience, Rutgers University, Newark, NJ USA

**Keywords:** Schizophrenia, Phencyclidine, Neurophysiology, Novel object recognition, Medial prefrontal cortex, Nucleus accumbens

## Abstract

The neural mechanisms underlying cognitive deficits in schizophrenia are poorly understood. Sub-chronic treatment with the NMDA antagonist phencyclidine (PCP) produces cognitive abnormalities in rodents that reliably model aspects of the neurocognitive alterations observed in schizophrenia. Given that network activity across regions encompassing medial prefrontal cortex (mPFC) and nucleus accumbens (NAc) plays a significant role in motivational and cognitive tasks, we measured activity across cortico-striatal pathways in PCP-treated rats to characterize neural enabling and encoding of task performance in a novel object recognition task. We found that PCP treatment impaired task performance and concurrently (1) reduced tonic NAc neuronal activity, (2) desynchronized cross-activation of mPFC and NAc neurons, and (3) prevented the increase in mPFC and NAc neural activity associated with the exploration of a novel object in relation to a familiar object. Taken together, these observations reveal key neuronal and network-level adaptations underlying PCP-induced cognitive deficits, which may contribute to the emergence of cognitive abnormalities in schizophrenia.

## Introduction

Schizophrenia is a devastating mental disorder characterized by positive, negative, and cognitive symptoms. Cognitive deficits are a hallmark of this condition, and understanding the mechanisms of these symptoms is of particular significance as they are highly predictive of long-term disease prognosis (Green 1996). Cognitive symptoms include deficits in working memory and behavioral flexibility (Forbes et al. 2009; Leeson et al. 2009), two processes of executive function that are essential for normal cognition. Both the Measurement and Treatment Research to Improve Cognition in Schizophrenia initiative (MATRICS) and the Cognitive Neuroscience Treatment Research to Improve Cognition in Schizophrenia group (CNTRICS) highlight cognitive deficits as a core feature of this disorder (Nuechterlein et al. 2008, 2009). These deficits are resistant to treatment and the neural mechanisms that underlie them are not well understood (an der Heiden and Hafner 2000; Riedel et al. 2006).

Dysfunction of the glutamatergic system, specifically *N*-methyl-d-aspartate (NMDA) receptor hypofunction, has been hypothesized to play a central role in schizophrenia pathogenesis (Kantrowitz and Javitt 2010; Olney et al. 1999). Thus, NMDA receptor antagonists, such as phencyclidine (PCP), mimic deficits in executive function and working memory in humans (Cosgrove and Newell 1991; Krystal et al. 1994). Sub-chronic treatment with an NMDA antagonist in rodents similarly produces deficits in cognitive tasks including novel object recognition (Bado et al. 2011; Grayson et al. 2015). Critically, PCP-induced deficits in rodents are still present after prolonged drug-free periods (1 week and longer) and thus reflect enduring functional changes rather than acute drug effects. PCP-pre-treated animals exhibit impaired performance on novel object recognition, spontaneous alternation, and pre-pulse inhibition, which are believed to model cognitive and negative symptoms of human schizophrenia (Castane et al. 2015; Grayson et al. 2015; Jorgensen et al. 2015). The mechanisms through which PCP produces behavioral impairments are largely unknown, but neurophysiological and immunochemical results point to the involvement of prefrontal cortical circuits (Kargieman et al. 2012; Piyabhan et al. 2013; Young et al. 2015).

Medial prefrontal cortex (mPFC) provides a major input to nucleus accumbens (NAc), a structure widely implicated in motivational and cognitive tasks (Bolding et al. 2012; Pezze et al. 2009). Specifically, prelimbic cortex is implicated in behaviors relevant to negative symptomatology, such as goal-directed behavior (Chudasama et al. 2001; Killcross and Coutureau 2003). Cortico-accumbal inputs play a role in cognitive function, such as working memory and attention, and a recent patient fMRI study showed synchrony deficits between mPFC and accumbens in schizophrenics (French and Totterdell 2003; Richter et al. 2015). Limbic structures including NAc have been hypothesized to underlie some of the behavioral abnormalities in schizophrenia and show abnormalities in patient brain scans (Grace 2000; van Erp et al. 2015). Consistent with this, NAc shell but not core was hypothesized to mediate some of the therapeutic effects of antipsychotics (Shilliam and Dawson 2005). In rats, acute NMDA receptor blockade causes abnormal local field potential oscillations in NAc (Goda et al. 2015). These observations suggest that deficits in cortico-accumbal network activity may underlie some aspects of compromised behavioral function (Bolding et al. 2012).

To address this here, we recorded single-unit activity in NAc shell and mPFC in awake, behaving rats pre-treated with PCP. Cognitive deficits in NMDA antagonist-pre-treated animals are widely studied using the novel object recognition (NOR) task (Grayson et al. 2015; Rajagopal et al. 2014), yet the neural dysfunction underlying NOR deficits is not well understood. Consistent with the hypothesized role of prefrontal cortex in negative symptomatology, rodent mPFC is implicated in working memory and visual recognition memory (Akirav and Maroun 2006; Yoon et al. 2008). Here we found that synchrony between mPFC and NAc was disrupted by PCP pre-treatment and overall activity in the NAc shell was reduced. In addition to this global deficit, we found that phasic responses in both structures in response to ongoing behavior were affected: PCP pre-treatment disrupted the increase in mPFC and NAc activity associated with novel object exploration in control animals.

## Results

### Sub-chronic PCP treatment reduced population firing rates in NAc shell

**Fig. 1:**
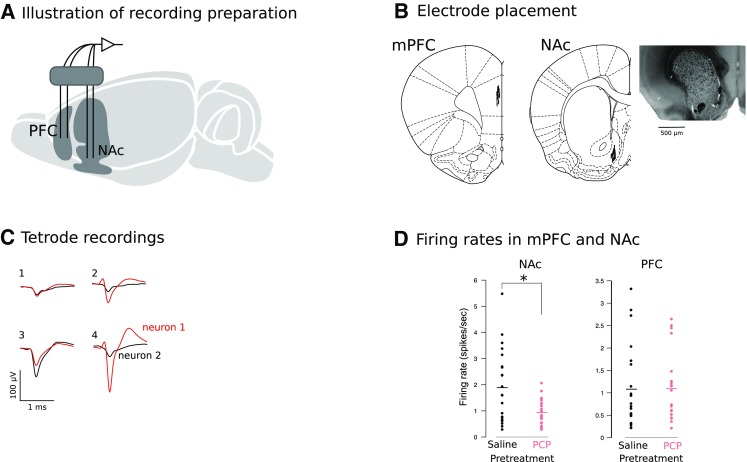
PCP pre-treatment reduces NAc single-unit activity. **a** Schematic illustration of the awake recording preparation. **b** Tetrode placements in mPFC (*left*) and NAc shell (*right*) including an example coronal section indicating vertical tract left by the recording electrode in NAc marked with an electrolytic lesion. **c** Illustration of tetrode-based spike sorting procedure. Four recording channels are shown from which two neurons were extracted. Spike shapes differed between units and across channels. **d** Effect of sub-chronic PCP pre-treatment on firing rates in single units recorded from NAc and mPFC. Data points represent individual units; horizontal lines represent group means. Sub-chronic PCP significantly reduced firing rates in NAc but not mPFC

To investigate cortical and NAc contributions to PCP-induced behavioral deficits, we recorded firing rates in single units recorded from mPFC and NAc shell in awake behaving rats pre-treated with either PCP or saline (Fig. 1a–c). Recordings were carried out during performance of a NOR task, and we tracked the activity of all units during the three 3-min trials of the task (pre-exposure, acquisition, retention). The effect of trial was statistically analyzed but was found to be non-significant. We recorded 41 mPFC single units (22 in PCP-pre-treated rats) and 39 NAc single units (19 in PCP-pre-treated rats). Medium spiny neurons (MSN) represent more than 90% of rat striatal and accumbal neurons, and unlike GABAergic interneurons are characterized by relatively low firing rates. We recorded units with low baseline activity (<6 Hz), and the firing rates we observed (see below) are consistent with previous studies (Barnes et al. 2005; Sharott et al. 2009). Similarly, in mPFC, firing rates were consistent with the cells being regularly spiking units (Bruno and Simons 2002). Consistent with previous work, we found no significant effect of PCP pre-treatment on mPFC firing rates [saline: 1.08 Hz ± 0.18 vs. PCP: 1.10 Hz ± 0.18 (mean ± SEM)] and no significant effect of trial (data not shown). However, sub-chronic PCP pre-treatment reduced putative MSN firing rates in NAc [saline: 1.89 ± 0.21 Hz vs. PCP: 0.93 ± 0.18 Hz] (Fig. 1d). This was supported by a significant effect of drug treatment [*F*(1, 37) = 7.28, *p* < 0.01]. To complement the unit-level analysis, we also calculated firing rate averages per animal and observed a structure (PFC vs. NAc) × drug group (PCP vs. vehicle) interaction [*F*(1, 20) = 2.183, *p* = 0.003] and no main effects. Following this up with LSD post hoc revealed significant reduction in firing rates in the NAc (*p* < 0.001) but not PFC (*p* = 0.577) of PCP-pre-treated animals confirming the main finding.

Additional burst and irregularity analyses showed spike timing characteristics consistent with previous work for both cortical and striatal neurons (Holt et al. 1996; Stern et al. 1997). Coefficients of variation [CV2 (Holt et al. 1996)] did not differ significantly between drug conditions or structures [NAc, 1.16 (SD = 0.12); mPFC, 1.17 (SD = 0.09)]. Burst detection was performed using Neuroexplorer based on the Poisson surprise method. The method which is well established for detecting burst activity in striatum (Estrada-Sanchez et al. 2015; Stanford and Gerhardt 2001) relies on comparing successive interspike intervals (ISIs) in the recorded spike train to a theoretical Poisson spike train with the same firing frequency. As done previously, we used a surprise threshold of 5 which estimates that a burst occurs ~150 times more frequently than would be expected in a Poisson spike train with the same mean firing rate (Estrada-Sanchez et al. 2015). Consistent with the single-unit data, there was a significant reduction in the number of bursts by PCP in the NAc [number of bursts per trial (3 min) for PCP: 5.31 ± 1.99 vs. saline: 9.11 ± 1.07; *p*=. 012] but not the mPFC (PCP: 5.67 ± 1.18 vs. saline: 6.60 ± 1.21; *p* = 0.583). There were no significant group effect on number of spikes per burst (mPFC: 13.66 ± 1.49; NAc 15.46 ± 1.38) or mean burst duration (mPFC: 2.43 ± 0.31; NAc 1.68 ± 0.29). These bursting parameter values are consistent with previous work in cortex and striatum (Estrada-Sanchez et al. 2015). The effect of trial was not significant.

### PCP pre-treatment results in abnormal cortico-accumbal synchrony

**Fig. 2:**
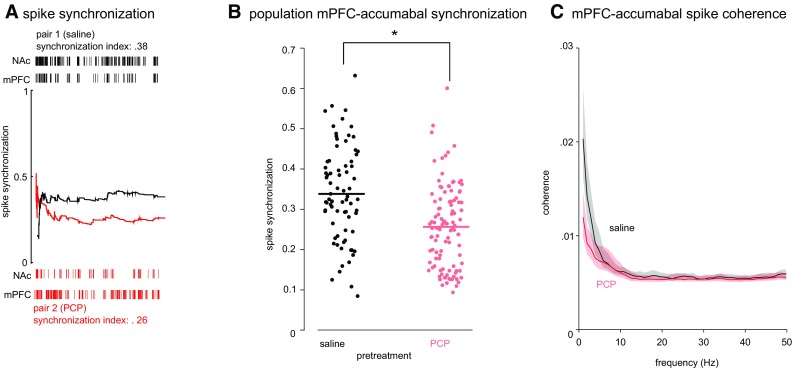
Cortico-accumbal synchrony is reduced in PCP-pre-treated animals. **a** Illustration of spike synchronization measure for two mPFC–NAc pairs recorded from a saline (*top*) and PCP-pre-treated (*bottom*) rat during the 3-min test trial of the novel object recognition task. The index is based on counts of quasi-simultaneous occurrences of spikes from a concurrently recorded pair, where the time lag used for calculating simultaneity is defined locally to allow for firing rate changes (see “Methods”). Spike trains are shown at the *top* and *bottom of the panel* and the cumulative normalized synchronization index is shown with a *black* and *red curve*, respectively. **b** Population data show significantly lower synchronization in PCP-pre-treated rats. Data points represent individual pairs; horizontal lines represent group means. **c** Average coherence across all mPFC–NAc spike pairs showed reduced coherence in the lower frequency bands. (*Error bands* represent a 95% bootstrap interval.)

To investigate spike synchrony, we calculated spike synchronization between 182 cortico-accumbens neuronal pairs (106 pairs from sub-chronic PCP-pre-treated animals). In the population, average spike synchronization between mPFC and NAc shell was significantly reduced by PCP pre-treatment [*F*(1, 727) = 38.37, *p* < 0.001; Fig. 2b]. The effect of trial was not significant suggesting that the disruption in cortico-accumbal synchronization may be relatively stable and independent of behavioral state at least in the NOR paradigm. Because the window we used to quantify synchronization is adapted to the firing rate (see “Methods”), it is unlikely that reducing NAc spiking activity in PCP-pre-treated animals may account for the significant reduction in synchronization observed here. To completely rule out this possibility, we recalculated synchronization based on reduced NAc spike trains (50% of spikes randomly sampled from the original spike trains) in the saline treatment group. Synchronization was still significantly reduced in PCP-pre-treated animals when based on this reduced dataset. To confirm the observation found on spike synchrony, we also analyzed mPFC–NAc synchrony using an independent spectrum-based measure: we found that average mPFC–NAc coherence across spike pairs was reduced by PCP pre-treatment (Fig. 2c; note non-overlapping 95% bootstrapped confidence intervals for the lower frequency bands).

### Sub-chronic PCP decreases novel exploration-related neural activity in mPFC and NAc shell

Because PCP-induced behavioral deficits include NOR and NOR performance requires intact mPFC (Barker et al. 2007), we were interested in the extent to which PCP pre-treatment affected neural responses on contact with the novel vs. familiar object. Because object exploration times were not normally distributed (*p* < 0.05, Shapiro–Wilk test, *W* = 0.862, *df* = 12), familiar versus novel object exploration times were compared with Wilcoxon signed-rank tests. As expected, this showed significant differences in exploration times in the saline (Wilcoxon *Z* score = 2.201; effect size: *r* = 0.64; *p* < 0.05), but not in the PCP-pre-treated rats (Wilcoxon *Z* score = 0.105; effect size: *r* = −0.03; *p* = 0.92). Median exploration times for the familiar and novel object were 29.9 versus 36.2 s in saline-pre-treated rats and 30.1 versus 30.8 s in PCP-pre-treated rats. Because non-parametric statistics are not available for mixed designs, we used bootstrapping to test the group effect: we calculated the median difference between novel and familiar exploration for both saline- and PCP-pre-treated animals. We then bootstrapped the difference between these two medians (saline minus PCP; Matlab command *bootci*, 2000 bootstrap samples), which yielded a 95% confidence interval of [4.1; 37.5] confirming the group effect. Further, we found no evidence that drug treatment affected the way the animals interacted with the object: we analyzed the amount of time each animal spent sniffing, touching, and approaching each of the two objects using the recorded video files. A mixed ANOVA (3 behavior types: sniffing, touching, or approaching × object: familiar vs. novel × drug treatment) produced no significant effects of either drug treatment or object or any interactions (*p*s > 0.10).

**Fig. 3:**
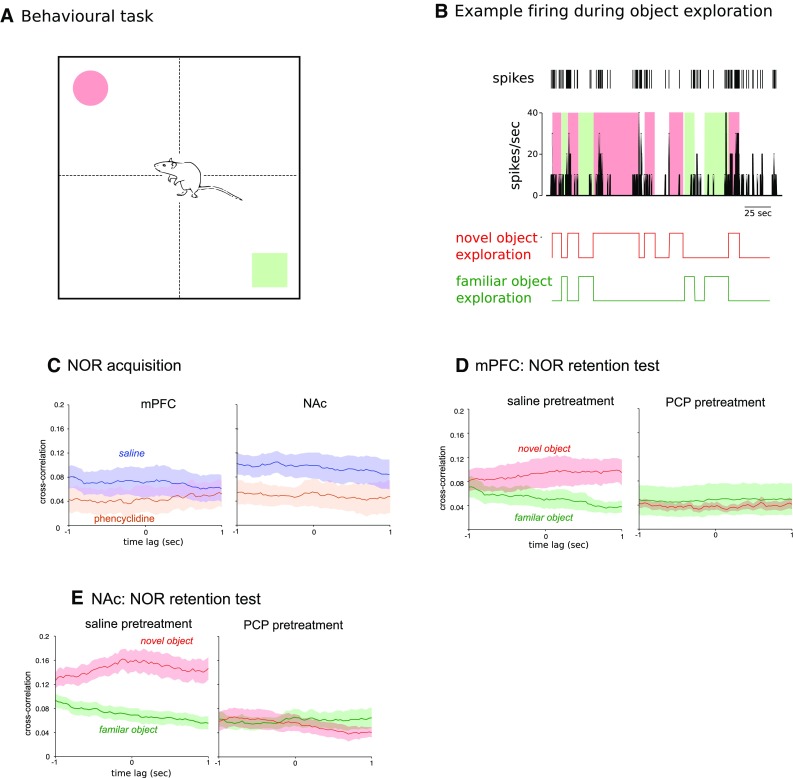
PCP pre-treatment disrupts mPFC and NAc activity associated with novel object contact in the novel object recognition task. **a** Illustration of testing procedure. The task measures interaction with a novel compared to a previously pre-exposed object, both placed in opposite corners of a testing arena. **b** Single-unit spike trains (*top*) were used to construct firing rate histograms (*middle*) which were aligned to ongoing exploration of the novel and familiar objects (*bottom*). **c** Cross-correlation between object exploration during the acquisition trial and spike activity in mPFC (*left*) or NAc (*right*) for PCP and saline-pre-treated animals. Cross-correlation between mPFC (**d**) or NAc (**e**) spike activity and exploration of the novel versus familiar object in the retention test. (*Error bands* represent SEM.)

To compare ongoing exploration times to spike activity, we quantified object contact from video recordings and aligned those to neuronal firing rate histograms from both mPFC and NAc shell (Fig. 3b). We calculated cross-correlations between object contacts and firing rate histograms. Because object contacts were coded as 1 versus 0 for no contact, the positive cross-correlation curves indicated higher exploration-related activity relative to no exploration across groups, an effect which during the retention trial was accentuated for the novel object compared to the familiar object after saline pre-treatment in both structures (Fig. 3d, e). On the other hand, PCP pre-treatment completely suppressed the association between spike response and novel object exploration in both NAc and mPFC. We then checked if this mirrored the acquisition trial during which both objects presented are novel. During acquisition exploration, cross-correlation averaged over the two novel objects was indeed lower for the PCP-treated animals and this effect attained significance in NAc (see non-overlapping error bands; Fig. 3c). We conclude that chronic PCP pre-treatment suppressed the relative enhancement of neuronal activity during exploration vs. no exploration for the novel object in both mPFC and NAc shell.

Finally, we calculated average firing rate (per structure) and synchrony values for each animal to investigate whether they correlate with object exploration times. The measures did not relate to either novel object or familiar object exploration (*p*s > 0.05). These null findings however are not surprising as they involve aggregation across time of both measures (neural and behavioral) with a corresponding decrease in power. This is in contrast to the temporally resolved analysis presented in Fig. 3, which does show a significant behavioral correlate of the spike activity.

## Discussion

PCP induces psychotic episodes in humans (Javitt and Zukin 1991), and its administration in rodents is widely used as an animal model of schizophrenia. Sub-chronic PCP treatment in rodents produces a number of behavioral abnormalities, which model cognitive and negative symptoms observed in schizophrenia. This study represents the first analysis of deficits in the cortico-accumbal circuit induced by sub-chronic NMDA antagonist administration in rats. As done by a number of different labs reporting on phencyclidine-induced deficits in rats, the current study used female animals (e.g., Martinez et al. 1999; Miyauchi et al. 2016; Sahin et al. 2016). This makes our results directly comparable to this previous work; importantly, however, previous studies also suggest female rats show higher vulnerability in the phencyclidine model (Snigdha et al. 2011), suggesting they are better suited for characterizing the deficits incurred by phencyclidine. Hormonal influences may affect sensitivity to PCP and this has been previously investigated in a rat model using acute PCP (Sutcliffe et al. 2008); however, in our study, both PCP- and saline-pre-treated animals were group-housed in the same holding room and tested in parallel. Thus, we do not suspect systematic differences in oestrous cycles to be a contributing factor for the differences we report here.

We found that repeated PCP treatment resulted in a tonic reduction in NAc shell single-unit firing rates and disrupted cortico-accumbal synchronization. These two regions are connected anatomically, and prelimbic cortex is implicated in behaviors relevant to negative symptomatology, such as goal-directed behavior and attention (Chudasama et al. 2001; Coutureau and Killcross 2003). Furthermore, nucleus accumbens shell but not core is hypothesized to mediate the therapeutic effects of antipsychotics (Shilliam and Dawson 2005). We also observed that phasic responses in both NAc shell and mPFC in response to ongoing behavior were affected: PCP disrupted the increase in mPFC and NAc neural activity, which was observed in vehicle-treated animals during exploration of a novel object. Consistent with the role of cortico-accumbal synchrony in memory and motivation, these neurophysiological deficits may drive widely reported cognitive abnormalities after sub-chronic NMDA antagonist treatment.

Previous studies by us and others have found impairments in visual working memory, pre-pulse inhibition, and NOR (Grayson et al. 2015; Mandillo et al. 2003; Sood et al. 2011). Consistent with these studies, here we observed a NOR impairment in PCP-pre-treated rats. Using an administration protocol, which produces reliable behavioral effects, we also observed a reduction in cortico-accumbal synchrony in PCP-pre-treated rats. These results are consistent with the role of PFC in schizophrenia and with previous work showing PCP-induced mPFC deficits. Thus, PFC is implicated in the neuropathology of schizophrenia with a notable increase in pyramidal cell density found in schizophrenic patients’ postmortem (Cullen et al. 2006) and abnormal cortical gamma-band oscillations (McNally et al. 2013; Uhlhaas and Singer 2010; Woo et al. 2010). In rodents, recent research shows disruption in single-unit responses in mPFC and abnormal synchrony after PCP treatment (Kargieman et al. 2007, 2012; Young et al. 2015). mPFC electrophysiological and neuroanatomical deficits in the sub-chronic PCP model suggest that abnormalities in a neural network encompassing mPFC may mediate novel object recognition deficits in this model (Castane et al. 2015; Young et al. 2015). The short retention intervals (1 min) tested in the NOR task in the PCP model are consistent with the role of mPFC in working memory (Grayson et al. 2007; Rosemann et al. 2010; Snigdha et al. 2011; Yoon et al. 2008). Interestingly, however, when longer retention delays are used (5 min or more), mPFC lesions do not impair object recognition (Barker and Warburton 2011; Sutcliffe et al. 2007). On the other hand, mPFC manipulations immediately after exposure impair long-term retention of the task (Akirav and Maroun 2006) further implicating mPFC on shorter retention timescales consistent with the role of the structure in working memory. Furthermore, lesion studies in both primates and rodents implicate mPFC in memory paradigms (Pezze et al. 2009; Rossi et al. 2009). Thus, prefrontal neurophysiological deficits observed in the PCP model may drive the behavioral abnormalities. Our results extend this by suggesting that abnormalities in cortico-accumbal projections may mediate some of the deficits. Consistent with the involvement of NAc, recent human imaging data suggest NAc abnormalities in schizophrenic patients (van Erp et al. 2015). In rats, one recent study reports altered local field oscillations in NAc in the methylazoxymethanol acetate (MAM) neurodevelopmental rodent model of schizophrenia (Goda et al. 2015). In drug-naïve animals, NAc single-unit activity increases in response to novelty during entry into a novel compartment (Wood and Rebec 2004) which is also consistent with our observations in the NOR task. Previous research implicating interactions between NAc and PFC in memory control supports the role NAc–PFC synchrony implicated in the current study. For example, a PFC–NAc disconnection significantly impaired auto-shaping in a stimulus-reward learning task, and attentional deficits in the 5-choice serial reaction time task produced by a PFC lesion are rescued by a dopaminergic antagonist in NAc (Parkinson et al. 2000; Pezze et al. 2009).

The deficits in mPFC–NAc communication observed here may involve more distributed circuits involving potentially hippocampus. Ventral hippocampal and mPFC projections to NAc show complex interactions, which were compromised in the MAM model, potentially involving ventral subiculum hyperactivity and mPFC hypoactivity (Belujon et al. 2014). NAc is a central structure for the integration of mPFC and hippocampal input. Interestingly, ventral hippocampal hyperactivity, as found in the MAM rodent model of schizophrenia (Lodge and Grace 2007), is proposed to disrupt NAc responses, providing a potential mechanism for the reduced NAc firing in the current study (O’Donnell et al. 1999). Also, hippocampal electrical high-frequency stimulation depotentiates the mPFC–NAc pathway (Goto and Grace 2005), and thus similarly plastic responses to hippocampal hyperactivity may underlie the reduced mPFC–NAc synchronization observed here. The precise systems interactions responsible for these effects must await further investigation. Interestingly, the MAM model results in some behavioral deficits which are similar to those reported in PCP-pre-treated rats, including impairments in reversal learning, and hypersensitivity to acute NMDA blockade (Belujon et al. 2014; Hradetzky et al. 2012). The involvement of distributed circuits is also suggested by the morphological abnormalities in limbic structures including anterior cingulate cortex and striatum observed in sub-chronic PCP-pre-treated rats (Barnes et al. 2014; Ingallinesi et al. 2014). Disruption in synchrony in the current study was observed between putative pyramidal, cortical and medium spiny neurons. Other neuromodulatory systems and/or striatal interneurons may mediate these effects. For example, prefrontal dopamine or NMDA blockade modulates NAc dopamine release (Del Arco and Mora 2008). Furthermore, NAc infusion of a dopamine antagonist has been found to rescue attentional deficits produced by prefrontal lesions (Pezze et al. 2009). Another potential source of modulation is NAc cholinergic interneurons, which modulate glutamatergic input as well as dopamine release in striatum (Gonzales and Smith 2015; Sofuoglu and Mooney 2009).

We also observed an increase in neuronal firing in mPFC and NAc shell associated specifically with novel object exploration in vehicle-treated animals in comparison to the familiar object consistent with previous studies showing novelty-induced activity in these structures recorded separately (Weible et al. 2009; Wood and Rebec 2004). Here this effect was abolished by PCP pre-treatment. These findings further support the conclusion that aspects of mPFC and NAc function, specifically related to memory recognition to novel and salient stimuli, are a central component of PCP-induced cognitive dysfunction.

## Methods

### Subjects

Twelve female Lister-Hooded rats, obtained from Charles River (Cambridge, UK), weighing between 225 and 250 g on arrival were housed in pairs prior to surgery on a 12-h reversed light–dark cycle (lights on at 1900 h) at an average temperature of 21 °C and humidity of 40–70%. All testing was carried out during the dark phase. Water and food (LabDiet 5LF5, PMI Nutrition Intl, Brentwood, MO) were freely available. The experiments were carried out under institutional ethics approval and appropriate project and personal license authority granted by the UK Home Office under the Animals (Scientific Procedures) Act 1986.

### Phencyclidine treatment

One week after arrival, rats received pre-treatment of PCP (Sigma-Aldrich, Gillingham, Dorset, UK; product nr. P3029) (2.0 mg/kg; *n* = 6) or saline (*n* = 6), in a final volume of 1 ml/kg i.p. twice daily for 7 days. Following PCP or saline treatment, the animals were given a 1-week drug-free period prior to surgery. The PCP dosing regimen was based on previous work demonstrating robust deficits in exploratory and memory paradigms and neurochemical deficits (Grayson et al. 2015; Sood et al. 2011).

### Tetrode implantation surgery

One week after PCP treatment, rats were anesthetised with 4% v/v isoflurane (Schering-Plough) in O_2_, and maintained between 2–3%. Immediately post induction, an injection of glycopyrronium bromide was administered (6–8 µg/kg; i.m.; Anpharm, Warsaw, Poland) to reduce respiratory tract secretions. The animal was mounted in a stereotaxic frame and the head was adjusted so that lambda and bregma were aligned on the same horizontal plane. To prevent corneal desiccation, Lacri-Lube Eye Ointment (Allergan, Westport, Ireland) was applied to the eyes. A homoeothermic heat pad (Harvard Apparatus, Boston, Massachusetts, USA) was used to maintain body temperature between 36 and 37 °C. Glucose (5%, 3 ml/h, s.c.) was given via an infusion pump (Intec, K.D, Scientific, Holliston, Massachusetts, USA) for the duration of the surgery.

A scalp incision was made along the midline, the periosteum was retracted, and 9–10 stainless steel anchoring screws (Morris Co., Southbridge, Massachusetts, USA, part number 0 × 1/8 flat) were affixed to the skull. A left-side craniotomy was then performed above mPFC and NAc shell. Implantation co-ordinates were as follows: +3.2 mm AP; 0.5 mm ML; −2 mm DV for mPFC, targeting prelimbic cortex; and +1.2 mm AP, 1.1 mm ML; −7 mm DV for NAc shell (Paxinos and Watson 1986). The medial of the two tetrodes per structure was targeted at these locations and distance between tetrode tips was minimal (~200 micron). The dura was incised and the tetrode array was advanced into the target structures using a stereotaxic holder. Two tetrodes were implanted in mPFC and two in NAc (Fig. 1a, b). Each tetrode was made of four 12 μm tungsten wires (H-Formvar insulation with Butyral bond coat, California Fine Wire Company, Brover Beach, CA) twisted together and heated to form a bundle. The tip of each wire was gold plated to reduce impedance to 150–400 kΩ. The tetrodes were threaded through a 0.17 mm outer diameter silica tube (SGE Analytical Science; Milton Keynes, UK) to increase stability and loaded into a microdrive (Versadrive, Neuralynx, Bozeman; Montana, USA) that allowed their independent movement. A silver wire inserted into the skull above the cerebellum served as a ground. The tetrodes were sealed with paraffin wax, and the implant was built up using layers of light-curing dental cement (Flowable Composite, Henry Schein; Gillingham, UK). Antibiotic ointment (Fuciderm; Uldum, Denmark) was applied to the wound and the skin was sutured. A non-steroidal anti-inflammatory (Carprieve, 5 mg/kg; S.C; Norbrook Laboratories Ltd; Corby, UK) was administered 2–3 h before recovery and twice a day for 5 days post surgery. An antibiotic (Baytril, 2.5%, 0.2 ml/kg; S.C., Bayer; Leverkusen, Germany) was given immediately after recovery and twice daily for 5 days after surgery. The animals were given a week to recover from the surgery before behavioral testing. Animals were handled daily and remained individually housed for the remainder of the experiment to prevent damage to the implants.

### Behavioral testing

PCP-induced cognitive deficits are commonly assessed using the novel object recognition (NOR) paradigm (Grayson et al. 2015). To verify drug manipulation and explore neurophysiological correlates of this behavioral deficit, we carried out recordings during NOR testing (Fig. 3a). Seven days post surgery, the electrodes were slowly advanced approximately 50 μm into the brain on three consecutive days. The tetrodes were advanced a further 50 μm approximately 30 min before each recording session. Electrophysiological recordings were obtained in an open-top black Plexiglas box (52 cm wide × 52 cm long × 40 cm high) placed within a sound-attenuated aluminum-plated chamber. The light intensity on the apparatus floor was maintained at approximately 15 Lux using indirect illumination provided by 8 LEDs evenly spread outside the testing box. The NOR protocol was based on well-established parameters (Grayson et al. 2015) and consisted of the following: After extensive habituation to the test box (20 min per day for three consecutive days), animals first underwent a short pre-exposure trial (3 min). No objects were placed in the arena during pre-exposure. This was followed by a 3-min acquisition trial, and a 3-min retention trial. All trials were separated by a 1-min inter-trial interval during which the animal was placed in a familiar holding cage (20 cm wide × 29 cm long × 38 cm high). During the acquisition trial, each rat was placed in the NOR chamber and exposed to two identical objects: the objects used were small glass jars or label-stripped food cans. On the retention trial, both objects were removed and one was replaced with an identical familiar copy and one with a novel object. The location of the novel object in the retention trial was randomly assigned for each rat. The heights of the objects were comparable (~10 cm) and they were heavy enough (475–500 g) not to be displaced by the animals. This was verified at the end of each session. Neurophysiological recordings were carried out throughout each testing trial.

All experiments were video recorded for subsequent behavioral analysis using a HD WebCam (1050 × 720p). Exploration times of each object in each trial were scored manually using Movie Maker (Microsoft Windows) with an on-screen millisecond stop watch. Video recordings were aligned to the neurophysiological data using a trigger programmed in the software Anymaze (San Diego Instruments, California, USA). Animals were deemed to be exploring the object when the head of animal was facing it within 2 cm or touching it with any part of its body except the tail: turning around or sitting on the object was not considered exploratory behavior.

### Electrophysiological recordings and data analyses

Rats were recorded during the three trials of the novel object recognition task (pre-exposure, acquisition, and retention) through a metal coil-wrapped headstage cable. We recorded from both PCP- and saline- pre-treated rats (2–4 animals/day; counterbalanced). Wideband signals were acquired continuously via an op-amp-based headstage amplifier (HST/8o50-G1-GR, 1× gain, Plexon Inc., Dallas, TX, USA), passed through a preamplifier (PBX2, 1000× gain; Plexon Inc., Dallas, TX, USA) and digitized at 40 kHz. For spike sorting, the raw signal was band-pass filtered, 300–3000 Hz, and spikes were sorted using the Matlab-based Wave_clus software to yield single-unit spike trains (Quiroga et al. 2004). Single units were detected by applying a threshold of 5× signal noise. Signal noise was estimated as the median absolute deviation of the band-passed signal (Rey et al. 2015). Spike sorting was achieved with super-paramagnetic clustering using a single parameter (‘temperature’), where in the super-paramagnetic regime, clusters of a relatively large size, corresponding to the different single units, are captured. All automatic detection thresholds and sorting solutions were examined individually and adjusted if needed. In addition to this, we inspected cross-correlograms and autocorrelograms of units obtained on the same wire as well as average cluster waveforms and ISI intervals for violations of a refractory period. PFC–NAc population synchrony was calculated as the average of synchrony values obtained across all neuronal pairs recorded from the two structures. While cross-correlograms are one possible method for analyzing simultaneously recorded spike trains, their use as a measure of spike timing synchronization has been disputed by some authors (Agmon 2012; Brody 1999). Here we applied two independent approaches to calculate synchrony. Firstly, we used a model-free spike synchronization algorithm based on counting the number of quasi-simultaneous occurrences of spikes from two concurrently recorded spike trains. The algorithm is described in detail in Quiroga et al. (2002) and the Matlab code is available online at: http://www.old.fi.isc.cnr.it/users/thomas.kreuz/Source-Code/Event-Sync.html. Synchronization values vary from 0 (no synchronization) to 1 (perfect synchronization) (Fig. 2a). The time lag used for detecting quasi-simultaneous occurrences is defined locally to allow for firing rate changes during recording (see Quiroga et al. 2002, Eq. 2):

$${\tau _{ij}} = \min \left\{ {t_{i + 1}^x} \right. - t_i^x,t_i^x - t_{i - 1}^x,t_{j + 1 - }^yt_j^y,t_j^y - \left. {t_{j - 1}^y} \right\}/2.$$

The lag *τ*_*ij*_ involves finding the minimum of the distances between a target spike *t*_*i*_ and the following one *t*_*i*+1_ as well as the target spike t_i_ and the preceding one *t*_*i*−1_ for both spike trains (*X* and *Y*) in a recorded pair (a total of 4 distances). In our sample, the median lag (across pairs and sessions) was equal to 25 ms. To complement this approach, we also calculated cross- and auto-spectrum-based spike coherence between PFC and NAc pairs (Halliday 2015) (Matlab code available online at http://www.neurospec.org). Comparisons between neural responses and ongoing object exploration were carried out using cross-correlations between firing rate histograms binned over 100 ms and continuous contact data scored as 0 (no contact) or 1 (for contact; Fig. 3b). The zero time point here refers to the center of the cross-correlogram between the two activity traces (i.e., the cross-correlation calculated at zero lag between the two channels). This approach essentially allowed us to measure the similarity between the two continuous signals (behavior and firing rate; compare Matlab command *xcorr*). A similar approach has recently been used successfully to assess the relationship between Purkinje cell activity and ongoing licking behavior in mice (Cao et al. 2012). All analyses were carried out using Neuroexplorer and Matlab (MathWorks, Natick, MA). Analyses of variance (ANOVA) and Wilcoxon signed-rank tests were performed using SPSS (IBM SPSS, Somers, NY, USA).

## References

[CR1] Agmon A (2012). A novel, jitter-based method for detecting and measuring spike synchrony and quantifying temporal firing precision. Neural Syst Circuits.

[CR2] Akirav I, Maroun M (2006). Ventromedial prefrontal cortex is obligatory for consolidation and reconsolidation of object recognition memory. Cereb Cortex.

[CR3] an der Heiden W, Hafner H (2000). The epidemiology of onset and course of schizophrenia. Eur Arch Psychiatry Clin Neurosci.

[CR4] Bado P, Madeira C, Vargas-Lopes C, Moulin TC, Wasilewska-Sampaio AP, Maretti L, de Oliveira  RV, Amaral OB, Panizzutti R (2011). Effects of low-dose D-serine on recognition and working memory in mice. Psychopharmacology (Berl).

[CR5] Barker GR, Warburton EC (2011). When is the hippocampus involved in recognition memory?. J Neurosci.

[CR6] Barker GR, Bird F, Alexander V, Warburton EC (2007). Recognition memory for objects, place, and temporal order: a disconnection analysis of the role of the medial prefrontal cortex and perirhinal cortex. J Neurosci.

[CR7] Barnes TD, Kubota Y, Hu D, Jin DZ, Graybiel AM (2005). Activity of striatal neurons reflects dynamic encoding and recoding of procedural memories. Nature.

[CR8] Barnes SA, Sawiak SJ, Caprioli D, Jupp B, Buonincontri G, Mar AC, Harte MK, Fletcher PC, Robbins TW, Neill JC, Dalley JW (2014). Impaired limbic cortico-striatal structure and sustained visual attention in a rodent model of schizophrenia. Int J Neuropsychopharmacol.

[CR9] Belujon P, Patton MH, Grace AA (2014). Role of the prefrontal cortex in altered hippocampal-accumbens synaptic plasticity in a developmental animal model of schizophrenia. Cereb Cortex.

[CR10] Bolding MS, White DM, Hadley JA, Weiler M, Holcomb HH, Lahti AC (2012). Antipsychotic drugs alter functional connectivity between the medial frontal cortex, hippocampus, and nucleus accumbens as measured by H215O PET. Front Psychiatry.

[CR11] Brody CD (1999). Correlations without synchrony. Neural Comput.

[CR12] Bruno RM, Simons DJ (2002). Feedforward mechanisms of excitatory and inhibitory cortical receptive fields. J Neurosci.

[CR13] Cao Y, Maran SK, Dhamala M, Jaeger D, Heck DH (2012). Behavior-related pauses in simple-spike activity of mouse Purkinje cells are linked to spike rate modulation. J Neurosci.

[CR14] Castane A, Santana N, Artigas F (2015). PCP-based mice models of schizophrenia: differential behavioral, neurochemical and cellular effects of acute and subchronic treatments. Psychopharmacology (Berl).

[CR15] Chudasama Y, Bussey TJ, Muir JL (2001). Effects of selective thalamic and prelimbic cortex lesions on two types of visual discrimination and reversal learning. Eur J Neurosci.

[CR16] Cosgrove J, Newell TG (1991). Recovery of neuropsychological functions during reduction in use of phencyclidine. J Clin Psychol.

[CR17] Coutureau E, Killcross S (2003). Inactivation of the infralimbic prefrontal cortex reinstates goal-directed responding in overtrained rats. Behav Brain Res.

[CR18] Cullen TJ, Walker MA, Eastwood SL, Esiri MM, Harrison PJ, Crow TJ (2006). Anomalies of asymmetry of pyramidal cell density and structure in dorsolateral prefrontal cortex in schizophrenia. Br J Psychiatry.

[CR19] Del Arco A, Mora F (2008). Prefrontal cortex-nucleus accumbens interaction: in vivo modulation by dopamine and glutamate in the prefrontal cortex. Pharmacol Biochem Behav.

[CR20] Estrada-Sanchez AM, Burroughs CL, Cavaliere S, Barton SJ, Chen S, Yang XW, Rebec GV (2015). Cortical efferents lacking mutant huntingtin improve striatal neuronal activity and behavior in a conditional mouse model of Huntington’s disease. J Neurosci.

[CR21] Forbes D, Culum I, Lischka AR, Morgan DG, Peacock S, Forbes J, Forbes S (2009). Light therapy for managing cognitive, sleep, functional, behavioural, or psychiatric disturbances in dementia. Cochrane Database Syst Rev.

[CR22] French SJ, Totterdell S (2003). Individual nucleus accumbens-projection neurons receive both basolateral amygdala and ventral subicular afferents in rats. Neuroscience.

[CR23] Goda SA, Olszewski M, Piasecka J, Rejniak K, Whittington MA, Kasicki S, Hunt MJ (2015). Aberrant high frequency oscillations recorded in the rat nucleus accumbens in the methylazoxymethanol acetate neurodevelopmental model of schizophrenia. Prog Neuro-psychopharmacol Biol Psychiatry.

[CR24] Gonzales KK, Smith Y (2015). Cholinergic interneurons in the dorsal and ventral striatum: anatomical and functional considerations in normal and diseased conditions. Ann N Y Acad Sci.

[CR25] Goto Y, Grace AA (2005). Dopamine-dependent interactions between limbic and prefrontal cortical plasticity in the nucleus accumbens: disruption by cocaine sensitization. Neuron.

[CR26] Grace AA (2000). Gating of information flow within the limbic system and the pathophysiology of schizophrenia. Brain Res Brain Res Rev.

[CR27] Grayson B, Idris NF, Neill JC (2007). Atypical antipsychotics attenuate a sub-chronic PCP-induced cognitive deficit in the novel object recognition task in the rat. Behav Brain Res.

[CR28] Grayson B, Leger M, Piercy C, Adamson L, Harte M, Neill JC (2015). Assessment of disease-related cognitive impairments using the novel object recognition (NOR) task in rodents. Behav Brain Res.

[CR29] Green MF (1996). What are the functional consequences of neurocognitive deficits in schizophrenia?. Am J Psychiatry.

[CR30] Halliday DM (2015). Nonparametric directionality measures for time series and point process data. J Integr Neurosci.

[CR31] Holt GR, Softky WR, Koch C, Douglas RJ (1996). Comparison of discharge variability in vitro and in vivo in cat visual cortex neurons. J Neurophysiol.

[CR32] Hradetzky E, Sanderson TM, Tsang TM, Sherwood JL, Fitzjohn SM, Lakics V, Malik N, Schoeffmann S, O'Neil MJ, Cheng TMK, Harris LW, Rahmoune H, Guest PC, Sher E, Collingridge GL, Holmes E, Tricklebank D, Bahn S (2012). The methylazoxymethanol acetate (MAM-E17) rat model: molecular and functional effects in the hippocampus. Neuropsychopharmacology.

[CR33] Ingallinesi M, Le Bouil L, Biguet NF, Do Thi A, la Cour CM, Millan MJ, Ravassard P, Mallet J, Meloni R (2014). Local inactivation of Gpr88 in the nucleus accumbens attenuates behavioral deficits elicited by the neonatal administration of phencyclidine in rats. Mol Psychiatry.

[CR34] Javitt DC, Zukin SR (1991). Recent advances in the phencyclidine model of schizophrenia. Am J Psychiatry.

[CR35] Jorgensen BP, Krych L, Pedersen TB, Plath N, Redrobe JP, Hansen AK, Nielsen DS, Pedersen C, Larsen C, Sorensen DB (2015). Investigating the long-term effect of subchronic phencyclidine-treatment on novel object recognition and the association between the gut microbiota and behavior in the animal model of schizophrenia. Physiol Behav.

[CR36] Kantrowitz JT, Javitt DC (2010). N-methyl-d-aspartate (NMDA) receptor dysfunction or dysregulation: the final common pathway on the road to schizophrenia?. Brain Res Bull.

[CR37] Kargieman L, Santana N, Mengod G, Celada P, Artigas F (2007). Antipsychotic drugs reverse the disruption in prefrontal cortex function produced by NMDA receptor blockade with phencyclidine. Proc Natl Acad Sci USA.

[CR38] Kargieman L, Riga MS, Artigas F, Celada P (2012). Clozapine reverses phencyclidine-induced desynchronization of prefrontal cortex through a 5-HT(1A) receptor-dependent mechanism. Neuropsychopharmacology.

[CR39] Killcross S, Coutureau E (2003). Coordination of actions and habits in the medial prefrontal cortex of rats. Cereb Cortex.

[CR40] Krystal JH, Karper LP, Seibyl JP, Freeman GK, Delaney R, Bremner JD, Heninger GR, Bowers MB, Charney DS (1994). Subanesthetic effects of the noncompetitive NMDA antagonist, ketamine, in humans. Psychotomimetic, perceptual, cognitive, and neuroendocrine responses. Arch Gen Psychiatry.

[CR41] Leeson VC, Robbins TW, Franklin C, Harrison M, Harrison I, Ron MA, Barnes TRE, Joyce EM (2009). Dissociation of long-term verbal memory and fronto-executive impairment in first-episode psychosis. Psychol Med.

[CR42] Lodge DJ, Grace AA (2007). Aberrant hippocampal activity underlies the dopamine dysregulation in an animal model of schizophrenia. J Neurosci.

[CR43] Mandillo S, Rinaldi A, Oliverio A, Mele A (2003). Repeated administration of phencyclidine, amphetamine and MK-801 selectively impairs spatial learning in mice: a possible model of psychotomimetic drug-induced cognitive deficits. Behav Pharmacol.

[CR44] Martinez ZA, Ellison GD, Geyer MA, Swerdlow NR (1999). Effects of sustained phencyclidine exposure on sensorimotor gating of startle in rats. Neuropsychopharmacology.

[CR45] McNally JM, McCarley RW, Brown RE (2013). Impaired GABAergic neurotransmission in schizophrenia underlies impairments in cortical gamma band oscillations. Curr Psychiatry Rep.

[CR46] Miyauchi M, Neugebauer NM, Oyamada Y, Meltzer HY (2016). Nicotinic receptors and lurasidone-mediated reversal of phencyclidine-induced deficit in novel object recognition. Behav Brain Res.

[CR47] Nuechterlein KH, Green MF, Kern RS, Baade LE, Barch DM, Cohen JD, Essock S, Fenton WS, Frese FJ, Gold JM, Goldberg T, Heaton RK, Keefe RS, Kraemer H, Mesholam-Gately R, Seidman LJ, Stover E, Weinberger DR, Young AS, Zalcman S, Marder SR (2008). The MATRICS Consensus Cognitive Battery, part 1: test selection, reliability, and validity. Am J Psychiatry.

[CR48] Nuechterlein KH, Luck SJ, Lustig C, Sarter M (2009). CNTRICS final task selection: control of attention. Schizophr Bull.

[CR49] O’Donnell P, Greene J, Pabello N, Lewis BL, Grace AA (1999). Modulation of cell firing in the nucleus accumbens. Ann N Y Acad Sci.

[CR50] Olney JW, Newcomer JW, Farber NB (1999). NMDA receptor hypofunction model of schizophrenia. J Psychiatr Res.

[CR51] Parkinson JA, Willoughby PJ, Robbins TW, Everitt BJ (2000). Disconnection of the anterior cingulate cortex and nucleus accumbens core impairs Pavlovian approach behavior: further evidence for limbic cortical-ventral striatopallidal systems. Behav Neurosci.

[CR52] Paxinos G, Watson C (1986). The rat in stereotaxic coordinates.

[CR53] Pezze MA, Dalley JW, Robbins TW (2009). Remediation of attentional dysfunction in rats with lesions of the medial prefrontal cortex by intra-accumbens administration of the dopamine D(2/3) receptor antagonist sulpiride. Psychopharmacology (Berl).

[CR54] Piyabhan P, Wetchateng T, Sireeratawong S (2013). Cognitive enhancement effects of *Bacopa monnieri* (Brahmi) on novel object recognition and NMDA receptor immunodensity in the prefrontal cortex and hippocampus of sub-chronic phencyclidine rat model of schizophrenia. J Med Assoc Thail.

[CR55] Quiroga QR, Kreuz T, Grassberger P (2002). Event synchronization: a simple and fast method to measure synchronicity and time delay patterns. Phys Rev E.

[CR56] Quiroga RQ, Nadasdy Z, Ben-Shaul Y (2004). Unsupervised spike detection and sorting with wavelets and superparamagnetic clustering. Neural Comput.

[CR57] Rajagopal L, Massey BW, Huang M, Oyamada Y, Meltzer HY (2014). The novel object recognition test in rodents in relation to cognitive impairment in schizophrenia. Curr Pharm Des.

[CR58] Rey HG, Pedreira C, Quiroga RQ (2015). Past, present and future of spike sorting techniques. Brain Res Bull.

[CR59] Richter A, Petrovic A, Diekhof EK, Trost S, Wolter S, Gruber O (2015). Hyperresponsivity and impaired prefrontal control of the mesolimbic reward system in schizophrenia. J Psychiatr Res.

[CR60] Riedel WJ, Mehta MA, Unema PJ (2006). Human cognition assessment in drug research. Curr Pharm Des.

[CR61] Rosemann M, Ivashkevich A, Favor J, Dalke C, Holter SM, Becker L, Racz I, Bolle I, Klempt M, Rathkolb B, Kalaydjiev S, Adler T, Aguilar A, Hans W, Horsch M, Rozman J, Calzada-Wack J, Kunder S,  Naton B, Gailus-
Durner V, Fuchs H, Schultz H, Beckers J, Beckers DH, Burbach JPH, Smidt MP, Quintanilla-Martinez L, Esposito I, Klopstock T, Klingenspor M, Ollert M, Wolf E, Wurst W, Zimmer A, de Angelis MH, Atkinson M, Heinzman U, Graw J (2010). Microphthalmia, parkinsonism, and enhanced nociception in Pitx3 (416insG) mice. Mamm Genome.

[CR62] Rossi AF, Pessoa L, Desimone R, Ungerleider LG (2009). The prefrontal cortex and the executive control of attention. Exp Brain Res.

[CR63] Sahin C, Doostdar N, Neill JC (2016). Towards the development of improved tests for negative symptoms of schizophrenia in a validated animal model. Behav Brain Res.

[CR64] Sharott A, Moll CK, Engler G, Denker M, Grun S, Engel AK (2009). Different subtypes of striatal neurons are selectively modulated by cortical oscillations. J Neurosci.

[CR65] Shilliam CS, Dawson LA (2005). The effect of clozapine on extracellular dopamine levels in the shell subregion of the rat nucleus accumbens is reversed following chronic administration: comparison with a selective 5-HT(2C) receptor antagonist. Neuropsychopharmacology.

[CR66] Snigdha S, Neill JC, McLean SL, Shemar GK, Cruise L, Shahid M, Henry B (2011). Phencyclidine (PCP)-induced disruption in cognitive performance is gender-specific and associated with a reduction in brain-derived neurotrophic factor (BDNF) in specific regions of the female rat brain. J Mol Neurosci.

[CR67] Sofuoglu M, Mooney M (2009). Cholinergic functioning in stimulant addiction: implications for medications development. CNS Drugs.

[CR68] Sood P, Idris NF, Cole S, Grayson B, Neill JC, Young AM (2011). PD168077, a D(4) receptor agonist, reverses object recognition deficits in rats: potential role for D(4) receptor mechanisms in improving cognitive dysfunction in schizophrenia. J Psychopharmacol.

[CR69] Stanford JA, Gerhardt GA (2001). Age-related changes in striatal function of freely-moving F344 rats. Neurobiol Aging.

[CR70] Stern EA, Kincaid AE, Wilson CJ (1997). Spontaneous subthreshold membrane potential fluctuations and action potential variability of rat corticostriatal and striatal neurons in vivo. J Neurophysiol.

[CR71] Sutcliffe JS, Marshall KM, Neill JC (2007). Influence of gender on working and spatial memory in the novel object recognition task in the rat. Behav Brain Res.

[CR72] Sutcliffe JS, Rhaman F, Marshall KM, Neill JC (2008). Oestradiol attenuates the cognitive deficit induced by acute phencyclidine treatment in mature female hooded-Lister rats. J Psychopharmacol.

[CR73] Uhlhaas PJ, Singer W (2010). Abnormal neural oscillations and synchrony in schizophrenia. Nat Rev Neurosci.

[CR74] van Erp TG, Hibar DP, Rasmussen JM, Glahn DC, Pearlson GD, Andreassen OA, Agartz I, Westlye LT, Hauvik UK, Dale AM, Melle I, Hartberg CB, Gruber O, Kraemer B, Zilles D, Donohue G, Kelly S, McDonald C, Morris DW, Cannon DM, Corbin A, Machielsen MW, Koenders L, de Haan L, Veltman DJ, Satterthwaite TD, Wolf DH, Gur RC, Gur RE, Potkin SG, Mathalon DH, Mueller BA, Preda A, Macciardi F, Ehrlich S, Walton E, Hass J, Calhoun VD, Bockholt HJ, Sponheim SR, Shoemaker JM, van Haren NE, Pol HE, Ophoff RA, Kahn RS, Roiz-Santianez R, Crespo-Facorro B, Wang L, Alpert KI, Jonsson EG, Dimitrova R, Bois C, Whalley HC, Mcintosh AM, Lawrie SM, Hashimoto R, Thompson PM, Turner JA (2015). Subcortical brain volume abnormalities in 2028 individuals with schizophrenia and 2540 healthy controls via the ENIGMA consortium. Mol Psychiatry.

[CR75] Weible AP, Rowland DC, Pang R, Kentros C (2009). Neural correlates of novel object and novel location recognition behavior in the mouse anterior cingulate cortex. J Neurophysiol.

[CR76] Woo TU, Spencer K, McCarley RW (2010). Gamma oscillation deficits and the onset and early progression of schizophrenia. Harv Rev Psychiatry.

[CR77] Wood DA, Rebec GV (2004). Dissociation of core and shell single-unit activity in the nucleus accumbens in free-choice novelty. Behav Brain Res.

[CR78] Yoon T, Okada J, Jung MW, Kim JJ (2008). Prefrontal cortex and hippocampus subserve different components of working memory in rats. Learn Mem.

[CR79] Young AM, Stubbendorff C, Valencia M, Gerdjikov TV (2015). Disruption of medial prefrontal synchrony in the subchronic phencyclidine model of schizophrenia in rats. Neuroscience.

